# Is it possible to predict improved diabetes outcomes following diabetes self-management education: a mixed-methods longitudinal design

**DOI:** 10.1136/bmjopen-2015-008781

**Published:** 2015-11-02

**Authors:** Caroline Huxley, Jackie Sturt, Jeremy Dale, Rosie Walker, Isabela Caramlau, Joseph P O'Hare, Frances Griffiths

**Affiliations:** 1 Warwick Medical School, University of Warwick, Coventry, UK; 2 Florence Nightingale Faculty of Nursing and Midwifery, King's College London, London, UK; 3 Successful Diabetes, Ipswich, UK; 4 Department of Psychology, Beaumont Hospital, Dublin, Ireland

**Keywords:** Self-management education, Predicting outcomes

## Abstract

**Objective:**

To predict the diabetes-related outcomes of people undertaking a type 2 Diabetes Self-Management Education (DSME) programme from their baseline data.

**Design:**

A mixed-methods longitudinal experimental study. 6 practice nurses and 2 clinical academics undertook blind assessments of all baseline and process data to predict clinical, behavioural and psychological outcomes at 6 months post-DSME programme.

**Setting:**

Primary care.

**Participants:**

–31 people with type 2 diabetes who had not previously undertaken DSME.

**Intervention:**

All participants undertook the Diabetes Manual 1:1 self-directed learning 12-week DSME programme supported by practice nurses trained as Diabetes Manual facilitators.

**Outcome variables:**

Glycated haemoglobin (HbA1c), diabetes knowledge, physical activity, waist circumference, self-efficacy, diabetes distress, anxiety, depression, demographics, change talk and treatment satisfaction. These variables were chosen because they are known to influence self-management behaviour or to have been influenced by a DSME programme in empirical evidence.

**Results:**

Baseline and 6-month follow-up data were available for 27 participants of which 13 (48%) were male, 22 (82%) white British, mean age 59 years and mean duration of type 2 diabetes 9.1 years. Significant reductions were found in HbA1c t(26)=2.35, p=0.03, and diabetes distress t(26)=2.30, p=0.03, and a significant increase in knowledge t(26)=−2.06, p=0.05 between baseline and 6 months. No significant changes were found in waist circumference, physical activity, anxiety, depression or self-efficacy. Accuracy of predictions varied little between clinical academics and practice nurses but greatly between outcome (0–100%). The median and mode accuracy of predicted outcome was 66.67%. Accuracy of prediction for the key outcome of HbA1c was 44.44%. Diabetes distress had the highest prediction accuracy (81.48%).

**Conclusions:**

Clinicians in this small study were unable to identify individuals likely to achieve improvement in outcomes from DSME. DSME should be promoted to all patients with diabetes according to guidelines.

**Strengths and limitations of the study**

- Thirteen diabetes-related clinical, behavioural and psychological outcomes were assessed for each participant.
- Data from quantitative and qualitative sources were used.
- Participants were new to diabetes self-management education.
- Selection bias regarding psychological outcomes was possible.
- In clinical practice, nurses have access to non-verbal clues and patient history in making assessments.

## Introduction

Diabetes Self-Management Education (DSME) is advocated for people with diabetes by major diabetes organisations across the developed world.^1–4^ Outcomes of DSME trials have been equivocal with most programmes demonstrating some effect on a range of outcomes including glycaemic control,^5^ ^6^ smoking cessation and illness beliefs,^7^ diabetes distress and self-efficacy,^8^ and quality of life.^6^ However, not all have demonstrated effects on the outcome of greatest clinical importance, namely glycaemic control.^7–9^ This has contributed to variability in healthcare professionals’ (HCPs) views of DSME, and the extent to which DSME is commissioned and delivered in health economies.^2^ UK National Institute for Health and Care Excellence (NICE) guidance advocates DSME (which in the UK is generally referred to as Diabetes Structured Patient Education) for all people with diabetes,^10–12^ with education being considered a key priority in the management of type 2 diabetes:

Offer structured education to every person and/or their carer at and around the time of diagnosis, with annual reinforcement and review. Inform people and their carers that structured education is an integral part of diabetes care. (ref. 12, p.6)

However, in 2012–2013, in England only 6.0% of all people with type 2 diabetes, and 16.7% of those newly diagnosed, had been offered a DSME programme.^13^ Uptake of DSME was even lower; only 1.6% of all people with type 2 diabetes, and 3.6% of the newly diagnosed, were recorded as attending DSME.^13^ In addition to ambivalence towards DSME, some HCPs have explained low referral rates to DSME by arguing that they can anticipate who will benefit from such programmes and will only refer those for whom advantages are perceived.^8^ ^14^ For example, HCPs have described reasons for low referrals based on their perceptions of patients’ ability to understand the content and awareness of the need for DSME.^14^ To address HCP ambivalence to refer patients to DSME, in England provision of DSME (Structured Education) became a Quality and Outcomes Framework^15^ item (pay for performance) in 2013 with the aim that more people will be referred. Nonetheless, referral does not guarantee uptake and attendance, and primary care professionals continue to have an important role in communicating the importance of DSME in improving a range of patient outcomes and encouraging attendance. The opportunity remains for HCPs to decide who to encourage based on their perceptions of likely patient benefit. Our objective was to assess the reliability of the argument offered by HCPs that they know whom to offer DSME. This paper presents findings from a broader study whose aims were to assess the feasibility of an enhanced Diabetes Manual programme. The research question addressed in this paper is: ‘Is it possible to predict the diabetes-related outcomes of people undertaking a type 2 DSME programme from their baseline and process data set?’.

## Methods

### Design

A mixed-method longitudinal experimental research design was employed between 2010 and 2012. Patients completed data collection when they consented to participate in the Diabetes Manual programme at the study baseline and at 6 months follow-up. Minimal important difference, that is, the smallest difference in outcome for a patient that is perceived to be meaningful,^16^ was used to measure change in outcomes for each individual. This paper presents a person-centred analysis.^17^ The detailed longitudinal data set enabled the construction of individual narratives examining how baseline variables are linked to specific outcomes.^18^

### The DSME intervention

The Diabetes Manual is an evidence-based 1:1 DSME programme for type 2 diabetes largely self-directed with support from practice nurses who have been trained as Diabetes Manual facilitators (DMF) to elicit behavioural changes and to provide psychological support. The Diabetes Manual consists of a workbook, relaxation audio components and a minimum of three face-to-face or telephone DMF contacts as preferred by the participant. The Diabetes Manual is designed to take 3 months to complete, involving approximately an hour a day for participants. Examples of how this hour may be spent includes reading the Diabetes Manual, taking physical activity, reading food labels, cooking a healthy meal, blood glucose monitoring or listening to the relaxation audio programme. The Diabetes Manual is evidence based^8^ ^19^ ^20^ and available for commissioning in the National Health Service (NHS) or direct purchase by people with type 2 diabetes.^21^

### Participants

Six practice nurses employed at general practices were recruited through the Primary Care Research Network and opportunistic sampling and two nurses were recruited from a hospital diabetes clinic. All were trained to become DMFs. The nurses each were asked to recruit up to 10 patients with type 2 diabetes, the ability to read English, glycated haemoglobin (HbA1c) >7.4% (57.4 mmol/mol) and who had not attended diabetes self-management education. Our sample size was based on the aims of the broader study in which we aimed to recruit 50 participants to give 80% confidence for assessing changes in diabetes management self-efficacy. After 2 months, two DMFs withdrew due to time pressures without recruiting patients. The remaining six DMFs consented 31 patients, four of whom subsequently withdrew.

### Procedures

Following completion of the training, DMFs arranged an appointment with participants to obtain informed consent, introduce them to the Diabetes Manual programme and collect baseline clinical assessments. The DMFs also gave the participants psychological outcome questionnaires and an accelerometer with instructions for use, which were collected from the participant 7 days later by the researcher. Within 2 months of recruiting their first patient, the DMFs were observed in two or three consultations by a trained facilitator for quality assurance (QA) purposes as per NICE guidance.^11^ A total of 17 QA consultations took place and were recorded. After their QA consultation, each patient took part in a brief interview with a researcher about their experiences in the consultation. Follow-up data collection took place 6 months postbaseline. Participants attended an appointment with their DMF where clinical measurements were taken. The psychological measures and accelerometer were sent to patients a week before their follow-up appointment. These measures were completed prior to their appointment. Finally, all participants took part in a follow-up interview to discuss their experiences of the DM programme, and any areas where the DSME had impacted on their diabetes management. The clinical, psychological, behavioural, process and demographic data known to impact on, or be impacted by DSME, and collected for the main study, represents the data set used to develop the outcome assessment framework.

### Outcome assessment framework

Our earlier Diabetes Manual randomised controlled trial (RCT)^8^ had found improvements in diabetes distress and self-efficacy. Subsequent meta-analyses^22^ had identified reduction in HbA1c with psychological intervention. We hypothesised therefore that with the addition of psychological care components to the Diabetes Manual, we would find clinically and statistically significant reductions in HbA1c. We further hypothesised that the more suboptimal the outcome in each patient, the greater the improvement would be although we did not develop hypotheses regarding DMF’s outcome prediction accuracy. We did not involve participants in making self-assessments regarding their anticipated outcomes of participating in the study. Each variable used in the outcome assessment framework is described, and inclusion justified, in table 1. Variables were included in the framework if they were (1) known to influence self-management behaviour (eg, ethnicity;^23^ depression^24^) or (2) known to have been influenced by a DSME programme in empirical evidence (eg, HbA1c;^5^ ^6^ diabetes distress^8^ ^25^). Data were collected at baseline and 6-month follow-up. Process indicator data, such as any patient change talk or value verbalised about the DSME, were collected during the QA process. Baseline measures and process indicators formed the data set used to ask the question, ‘Based on this patient’s data do I think that engaging with DSME will result in any changes in HbA1c, waist circumference, exercise levels, anxiety, depression and distress in 6 months’ time for this patient?’ With each patient and each outcome, the DMFs made a prediction through recording one of three expectations (1) the outcome would improve by a minimally important difference,^16^ (2) the outcome would deteriorate or (3) there would be no change. Specifically for prediction purposes, data were presented in a table format alongside clinical guidelines relevant to each outcome. All data in the framework could be available to nurses during routine consultations if they chose to access the information.

**Table 1:** Variables within the predictive framework

Framework data and, for outcomes only, MID	Measure, cut-offs as appropriate and completion mechanisms	Justification for inclusion
Demographic data	IMD: participant's home postcode was used to identify IMD ward. Deprived areas are those ranked lower than 6562 (the 20% most deprived wards in the UK)^34^ Ethnicity: self-report Gender, age, time since diagnosis and occupation are not known to be related to DSME effects so were not included in the framework	Deprivation is linked to less successful management of type 2 diabetes^35^ ^36^ Ethnicity is linked to type 2 diabetes prevalence^23^ and poorer self-management/diabetes outcomes^37^
Knowledge of diabetes	RDKS^37^ is a 19-item multiple choice scale assessing diabetes-related knowledge. Correct responses are coded as ‘1’ and incorrect responses are coded as ‘0’, and these are summed to give a possible score between 0 and 19	Knowledge is modifiable by intervention and people with low knowledge at baseline might expect to increase their knowledge, and subsequently improve their behavioural outcomes, following DSME^38^
Self-efficacy	DMSES tool is a 15-item scale that has been validated with UK populations.^37^ ^39^ Items are scored on a 0–10 Likert-type scale to indicate how confident they are at the task described. Responses are summed, giving possible self-efficacy scores between 0 (no self-efficacy) and 150 (very high self-efficacy)	Self-efficacy is modifiable by DSME.^8^ People with low self-efficacy might be expected to raise this following DSME
Diabetes distress The MID=half a SD.^40^ The SD was 20.84, therefore the MID=10 scale points	PAID^41^ is a 20-item scale measuring emotional functioning relating to diabetes, with each item scored on a 5-point Likert-type scale (0–4). Responses are summed and multiplied by 1.25 to give an overall score between 0 and 100. In order to categorise baseline levels of distress, PAID scores were categorised as either high distress (over 40 scale points), medium distress (20–40), or no distress (under 20)	Diabetes distress is modifiable by DSME.^8^ ^25^ People with high distress might be expected to have lower diabetes distress following DSME
Depression MID=1.5 scale points.^42^ Anxiety MID=1.5 scale points^42^	HADS^43^ is a 4-point Likert-type scale to indicate the extent of 14 anxious and depressive feelings over the past week. Responses are coded from 0 to 3 and the total is computed for each subscale (giving total scores between 0 and 21). Scores over 8 indicate clinical levels of anxiety/depression.^44^ The HADS has been used and validated with diabetic populations^45^	Depression is known to compromise self-management efforts^24^ and so it is likely that no changes in depression or key self-management outcomes will be seen in someone with depression
HbA1c MID=0.5%^40^	The clinical cut-off for uncontrolled diabetes, and for participation in the study, is 7.4% or 57 mmol/mol^46^	DSME has been shown to have an effect on HbA1c, and this is a key clinical marker of disease control^5^ ^6^
Waist circumference MID=5%^47^	For white and black men waist measurement should be below 94 cm, for Asian men it should be below 90 cm, and for all women it should be below 80 cm^48^	Waist circumference is a better predictor of health, and particularly type 2 diabetes, than is overall weight or BMI^49^ ^50^
Physical activity MID=an increase of 2500 steps per day.^51^	Yamax Powerwalker accelerometer was used for 3 days (including one weekday and one weekend day) to record (1) the number of steps (2) the number of kilometres walked and (3) number of calories burned. Data from the accelerometer was averaged for the 3 days. The recommended average steps per day is 10 000^51^	DSME focus on physical activity was high so the potential for 2500 step increase was change easy to observe
Change talk: changes made	Process measure identified during QA audio-recordings and mid-point interviews for 17/27 participants. A brief description of changes already made since starting the DSME was included. For example, patient 1 said: “I go to the gym three times a week now […] which I haven't done for about 20 years”	Patient-led change talk indicates readiness to initiate/sustain behaviour changes^52^
Change talk: changes planned	Process measure identified during QA audio-recordings and mid-point interviews for 17/27 participants. A brief description of changes planned during the QA consultation was included. For example, patient 24 said that she planned to increase her exercise so that she made herself out of breath: “Just when I do walk to step it up, yes to make sure to stop walking on the flat and taking it nice and easy, just to pick a few hills and go for it [laughs]. […] just make myself out of breath [for] more than five or ten minutes”	Patient-led change talk indicates readiness to initiate/sustain behaviour changes^52^
Treatment satisfaction with DSME	Process measure identified during QA audio-recordings and mid-point interviews for 17/27 participants. A brief description of comments made about the DSME was included. For example, patient 4 was very positive about the DSME: “I'm feeling actually much better and after going through my manual I felt it was quite informative…I enjoyed reading it”	Assessment of treatment satisfaction of an indicator of usefulness to participant

BMI, body mass index; DMSES, Diabetes Management Self-Efficacy Scale; HADS, Hospital Anxiety and Depression Scale; HbA1c, glycated haemoglobin; IMD, Index of Multiple Deprivation; MID, minimal important difference; PAID, Problem Areas In Diabetes Scale; RDKS, Revised Diabetes Knowledge Scale; QA, Quality Assurance.

### Methods of predicting outcome

Two clinical academic research team members (JS, a nurse and FG, a general practitioner (GP)) developed and pilot tested the prediction method using the outcome assessment framework. They independently examined individual patient baseline and process data and made predications pertaining to 6-month follow-up outcome. This iterative process was piloted with seven patients and then applied with the study population. Following individual predictions, discussion took place to reach agreement. On a separate occasion, six DMFs used the framework to predict outcomes on each other's anonymised patient data (ie, participants not known to them) in a recorded focus group. Patient data were randomly allocated to individual DMFs, ensuring that each DMF examined a unique subset of different patients, and that all patients’ data were examined by two different DMFs. Initially the DMFs made individual predictions for their own subset of 9 patients, then collectively they discussed and made predictions for 14 patients. Therefore, each participant data set was individually assessed by two DMFs (different for each patient) to produce specific predictions and a brief outcome narrative (see table 2, eg, data and predictions). Once all predictions were completed the 6-month follow-up data were entered into the framework to enable analysis. The standard of assessment for determining a positive change in outcomes for each patient was the minimally important difference unique to each outcome (as listed in table 1). During analysis, where no prediction was given for change or stability, this was classed as ‘no change’ predicted. Where there were disagreements about the prediction, this was noted and the prediction made by the majority was used in analysis. Accuracy is described per patient (ie, how many of the 6 outcome predictions per patient were accurate), and per outcome (ie, what percentage of the 27 predictions made for each outcome were accurate).

**Table 2:** Example prediction framework data

Patient	Guidelines	Patient 3 Data	Patient 3 Predictions	Patient 24 Data	Patient 24 Predictions
IMD	Deprived areas <6437.6	13 349		18 942	
Ethnicity		Indian		White British	
HbA1c	<7.4% or 57.4 mmol/mol	11.10% 97.8 mmol/mol	Reduced (baseline measurement is high so there is scope for change)	12.1% 108.7 mmol/mol	Reduced (had baseline been a bit lower no change would have been predicted because current dietary changes are not substantial and she is already doing enough exercise)
Waist	94 cm white/black men; <90 cm Asian men; <80 cm women	96.52 cm	None	114	Reduced
Exercise	>10 000 steps per day	4872	Increase (he talks about increasing his exercise)	10 700.67	None (already exercising enough)
Anxiety	Clinical limit >8	3	None	6	None
Depression	Clinical limit >8	3	None	0	None
Diabetes distress	>40 high distress; 20–40 moderate distress; <20 no distressed	53.75	Reduced (baseline measurement is high so there is scope for change)	10	None
Self-efficacy	>101 high; 51–100 moderate; <50 low	120		96	
Knowledge		13		14	
Change talk: changes made		Eats a healthy diet (mainly vegetarian, no alcohol, healthy food). Eats smaller portions. Has started walking more		Cut down on butter and fat in diet. Has started walking more	
Change talk: changes planned		To increase exercise (walking, cycling, swimming), and find time for it		To increase her exercise—walk enough to get out of breath	
Treatment satisfaction		Very positive about DSME		DSME is a bit repetitive at times	

DSME, Diabetes Management Self-Efficacy; HbA1c, glycated haemoglobin; IMD, Index of Multiple Deprivation.

## Results

Quantitative data on clinical, psychological and behavioural outcomes were collected at baseline from 30 patients and at 6 months from 27 patients. Of the latter, 13 (48%) were male, 22 (82%) were white British, ages ranged from 39 to 81 years (mean 59.2 years) and duration of type 2 diabetes was 3 months to 34 years (mean 9.1 years). See table 3 for participant clinical and psychological characteristics at baseline and follow-up. Among the whole group, there were significant reductions in HbA1c% t(26)=2.35, p=0.03, and diabetes distress t(26)=2.30, p=0.03, and a significant increase in knowledge t(26)=−2.06, p=0.05 between baseline and 6 months (see table 3). No significant changes were found in waist circumference t(26)=−0.43, p=0.67; physical activity t(26)=0.99, p=0.33; anxiety t(26)=−1.39, p=0.18; depression t(26)=−0.38, p=0.71; or self-efficacy t(26)=−1.83, p=0.08.

**Table 3:** Clinical and psychological characteristics of participants

	Baseline (N=30)		6-Month follow-up (N=27)		Difference between baseline and follow-up
	Range	Mean (SD)	Range	Mean (SD)	t
HbA1c (%)	7.10–13.60	9.20 (1.92)	5.60–13.30	8.40 (1.90)	2.35 (p=0.03)*
HbA1c (mmol/mol)	46.00–125.00	76.19 (21.90)	38.00–122.00	68.96 (20.90)	1.79 (p=0.09)
Waist	77.00–148.00	109.72 (15.12)	88.40–160.00	110.60 (16.34)	−0.43 (p=0.67)
Steps	106.00–17 223.67	5985.77 (3971.09)	12.67–16 593.00	5469.88 (3923.11)	0.99 (p=0.33)
Diabetes distress	0.00–83.75	22.82 (21.35)	0.00–70.00	16.71 (17.39)	2.30 (p=0.03)*
Anxiety	0.00–15.00	5.26 (4.37)	0.00–13.00	4.37 (3.61)	1.39 (p=0.18)
Depression	0.00–9.00	3.07 (2.43)	0.00–13.00	3.26 (2.98)	−0.38 (p=0.71)
Knowledge	7.00–20.00	14.37 (2.92)	9.00–22.00	15.67 (3.20)	−2.06 (p=0.05)*
Self-efficacy	62.00–146.54	104.66 (22.66)	75.00–157.00	113.00 (19.84)	−1.83 (p=0.08)

HbA1c, glycated haemoglobin.

*Significant p value.

### Researcher predictions (pilot)

The accuracy of the researcher predictions for change in the key clinical, behavioural and psychological outcomes for the seven pilot patients was examined. Accuracy of predictions ranged from no accurate outcome predictions to all six outcome predictions accurate. Accuracy of predictions per outcome varied from 42.86% to 71.43% (ie, of the seven predictions made for each outcome (one for each patient), between three and five were accurate). Prediction agreement between the two researchers was 96%.

### DMF predictions

The accuracy of the DMF predictions for key clinical, behavioural and psychological outcomes for the whole data set was examined. Although there were disagreements in individual predictions, an overall reduction in HbA1c was predicted for all patients. Change was less widely predicted for other variables. Clinically important outcomes not captured by clinical, psychological and behavioural data but revealed during interview include dietary changes and reduction in alcohol intake (n=7), increased medication adherence (n=1) and smoking cessation (n=1).

### Accuracy per patient

Accuracy of the six outcome predictions per patient ranged from one to six, with the median and mode being four out of six predictions. There were between one and six prediction agreements per patient, with an average of 4.12 prediction agreements in outcomes per patient. Overall, prediction agreement for the DMFs was 68%.

### Accuracy per outcome

Accuracy for predictions by outcome varied from 44.44% for HbA1c to 81.48% for diabetes distress. The median and mode accuracy was 66.67%. Table 4 shows the nature of the change predicted for each outcome and the percentage of patients in which changes were predicted for each outcome, the nature of actual change observed and the percentage of accurate predictions made. Many predictions were correct because no change was predicted and this was accurate.

**Table 4:** DMF predictions as to the impact of the DSME on clinical outcomes, and the accuracy of these predictions

	HbAc1	Waist	Exercise	Distress	Anxiety	Depression
Direction of predicted change (n=number of patients for which this change was predicted)	Reduced=27 No change=0	Reduced=6 No change=21	Increased=9 No change=18	Reduced=6 No change=21	Reduced=4 No change=23	Reduced=1 No change=26
Percentage of patients for which change was predicted	100.00	22.22	33.33	22.22	14.81	3.70
Total number of agreements in predictions*	12	19	13	17	23	26
Direction of actual change (n=number of patients for which this change was observed)	Reduced=12 No change=15	Reduced=4 No change=23	Increased=2 No change=25	Reduced=7 No change=20	Reduced=8 No change=19	Reduced=8 No change=19
Per cent of accuracy of predictions	44.44	70.37	62.96	81.48	62.96	74.07

*Disagreements were counted when one person or more disagreed with the majority prediction for the patient.

DMF, Diabetes Manual Facilitator; DMSE, Diabetes Self-Management Education; HbA1c, glycated haemoglobin.

## Discussion

### Summary of main findings

Findings from this study identified that accuracy for predicting change in HbA1c by the nurses was low with their accuracy for reductions in diabetes distress higher. We found that the majority of accurate predictions related to an anticipation of no change (ie, the nurses thought the patients 6-month outcomes would not change) in relation to waist circumference, physical activity levels, anxiety and depression. Overall, there was greater accuracy in predictions about lack of change, than in identifying individuals who would achieve improvement in outcome. Predictions for change were made most frequently for measures that showed the greatest room for change (ie, those outcomes in those patients with high baseline scores). The Diabetes Manual DSME programme has continued to demonstrate improvements in glycaemic control and diabetes distress in this new population.^8^ High HbA1c was an inclusion criterion for participation in the study and there was consistency in the prediction that the DSME intervention would reduce this, suggesting that health professionals can find it difficult to predict in what ways people will benefit from DSME. Health professionals appear to believe that their patients will change (optimistic bias) where scope for change is evident, and where they believe in the efficacy of their treatment endeavours. Prediction agreement within the group of nurses was much lower than for the researchers. This could indicate the lack of experience in undertaking this exercise in contrast to the researchers who had developed the process (so were more practiced in applying it). It was notable that, where available, the qualitative data were heavily drawn on by the nurses to inform predictions. Qualitative evidence was used to provide insight into underlying motivations for behaviour change and attitudes towards the intervention, consistent with the narrative development process for narratives exploring how baseline variables are linked to specific outcomes.^18^

### Strengths and limitations of the study

The research intervention used a tried and tested DSME programme which enabled the DMFs to focus on the research questions presented and not on DSME programme evaluation. The nurses were aware of the earlier RCT findings which may have influenced their decision-making. This research benefits from the assessment of a wide range of clinical and psychological measures, so that the anticipated impact of DSME on a variety of outcomes could be assessed. However, it is limited by the lack of a measure of eating habits. This is a key behavioural change targeted by the DSME, and which has the potential to significantly improve HbA1c.^26^ The baseline characteristics show that our participants’ anxiety and depression levels were low, so few changes were expected. This may indicate a participant selection bias, as people with depression could have been less likely to consent, or be offered the opportunity, to participate in the study. Several outcomes in our framework were self-report, and completing these could have been interventional in themselves by causing the participant to reflect on their mood, coping, confidence or knowledge. The DMFs were all experienced at working with people with type 2 diabetes, and so were able to draw on their clinical experience of this patient group when making predictions. The process and outcome narratives were, however, produced in a fairly artificial setting. In consultations, health professionals often have prior knowledge of their patients and draw on non-verbal data to make their assessments. Despite this, the health professionals did articulate why they would expect particular outcomes, making explicit the clinical evaluation process. In relation to study eligibility criteria, the DMFs struggled to first identify, and then recruit, patients who had not previously attended locally offered DSME and who could read English. This could indicate that patients in the settings served by these nurses had already been extensively offered DSME in contrast to the national average of 6.0%.^13^

### Comparison with wider literature

Nurses tend to draw heavily on their experience when interpreting the different sources of information available during routine consultations experience.^27^ ^28^ In this study, the DMFs used their experience to make inferences from the available data. Such inferences demonstrate how previous clinical experiences inform (or even bias) current evaluations.^29^ ^30^ Physicians’ clinical experience and knowledge have long been viewed as the ‘quintessential skills’ that they have to offer (ref. 31, p.657). Used alone, however, professional opinion has been described as the ‘least reliable and valid form of evidence’ on which to base clinical decisions (ref. 30, p.232), below hierarchies of research evidence. One systematic review found that physicians with more experiential knowledge were less likely to adhere to appropriate standards of care, a finding which they describe as inconsistent with the notion that experience enhances knowledge and skills, leading to better patient care.^32^ Other authors have claimed that experienced clinicians form hypothesises and diagnostic plans more quickly and to a higher standard than inexperienced clinicians.^33^ The benefit of clinical experience in making evaluations and decisions is somewhat contested then. In this study, where nurses had to make clinical judgements without physically seeing a patient, they had few other resources to draw on other than their experience with similar patients. This wider literature and the findings of this study have implications for the training of clinicians who refer people to DSME. If this study were repeated with GPs the findings may have been different.

## Conclusions

Our research indicates that while clinicians draw on their extensive clinical experience in assessing the benefit of DSME, in our relatively modest group of 6 practice nurses and outcome data on 27 people with diabetes, it was not possible for them to reliably and accurately determine outcomes utilising 10–12 pieces of data per person on which to make these assessments. Our results indicate that all people with diabetes should continue to be offered DSME programmes according to national^2^ ^10^ ^11^ and international guidelines.^1^ ^3^ ^4^ Furthermore, research should explore the clinical decision-making process, to make explicit the process through which clinicians make judgements on the potential benefit (or not) of DSME for different patients. Further exploration of this topic could highlight how clinical experience is used to interpret data within current situations, and the outcome this has for the patient in their access to care.
